# Current Concepts in the Management and Treatment of Spinal Metastases—A Systematic Literature Review

**DOI:** 10.3390/cancers17081296

**Published:** 2025-04-11

**Authors:** Adriana Palacio Giraldo, Verena Dammerer, Johannes Neugebauer, Gianpaolo Leone, Melanie Schindler, Dietmar Dammerer

**Affiliations:** 1 Department of Orthopedics and Traumatology, Karl Landsteiner University of Health Sciences, Dr. Karl-Dorrek-Straße 30, 3500 Krems, Austria; 2 Division of Orthopedics and Traumatology, University Hospital Krems, Mitterweg 10, 3500 Krems, Austria; 3 Regional Hospital Scheibbs, Eisenwurzenstraße 26, 3270 Scheibbs, Austria

**Keywords:** spinal metastasis, metastatic spinal disease, treatment options, management algorithm

## Abstract

Background: The advent of novel cancer therapies has significantly improved both life expectancy and quality of life for cancer patients. However, this has also led to an increased incidence of metastases, particularly spinal metastases, which pose a significant clinical challenge due to their potential to compromise spinal stability, mobility and neurological function. Objective: The aim of this study is to identify current symptom scores, determine the diagnosis, evaluate treatment modalities, and review prognostic scores and management algorithms for spinal metastases to aid in therapeutic decision making. Methods: A systematic literature review was conducted in PubMed focusing on available full-text review articles published in English between 1 January 2012 and 1 July 2022. The search terms were “spinal metastasis OR spinal metastases OR spine metastasis OR spine metastases AND treatment OR management”. Results: Several treatment algorithms, including NOMS (neurology, oncology, mechanical stability, and systemic disease) and LMNOP (localization, mechanical instability, neurology, oncology), have been proposed to guide clinical decision making. However, the development of prognostic scores that incorporate the impact of newer treatment modalities remains an important area of research. Conclusions: Effective management of spinal metastases requires a multidisciplinary approach, with emphasis on early diagnosis. Treatment decisions are individualized. Further research is essential to refine treatment strategies and improve patient outcomes.

## Simple Summary

This literature review focuses on the symptoms, diagnosis and treatment of spinal metastases, which represent a late complication of the primary tumor. The main treatment goals for spinal metastases include local control, pain relief, improvement or prevention of neurologic symptoms, and maintenance of stability. Magnetic resonance imaging is considered the gold standard for diagnosing spinal metastases, although alternative imaging modalities are also described. Treatment options include systemic therapies, radiotherapy, and surgical techniques for metastatic lesions. The combination of stereotactic body radiotherapy with surgical spinal decompression of the spine is an evolving treatment approach with promising results. Several treatment management algorithms, including the NOMS (neurology, oncology, mechanical stability, and systemic disease), and LMNOP (localization, mechanical instability, neurology, oncology) algorithms, have been proposed to aid in the decision-making process regarding individual treatment modalities. The primary factors influencing treatment decisions are primary tumor histology and life expectancy.

## 1. Introduction

Cancer is currently the second leading cause of premature death among noncommunicable diseases worldwide, after cardiovascular disease (CVD) [1,2]. Depending on the quality of the health care system, survival rates are increasing significantly due to medical advances [1]. Studies have shown declines in premature deaths from cancer ranging from approximately 20 to 30%, respectively, from 2000 to 2019 [2]. The increasing incidence of cancer has been attributed to an aging population and improvements in cancer treatment, leading to longer survival. This increases the likelihood of being diagnosed with metastatic disease, including spinal metastases [1,2,3,4,5]. Metastases are most commonly found in the liver, lungs, and bone tissue [3,4,6].

The spine is the most common site of bone metastasis, accounting for approximately 69%, followed by the pelvis (41%), proximal femoral metaphysis (25%), and skull base (14%) [3]. In comparison, primary spinal tumors are about 20 times less common [7].

According to the literature, around 40% of patients with metastatic cancer will develop spinal metastases during the course of their disease, but autopsies have shown that 70% of patients diagnosed with cancer have evidence of spinal metastases. Despite the high incidence, only 10–20% of these patients will become symptomatic [5,6,8,9,10]. The thoracic spine is most commonly affected (70%) due to its larger number of vertebrae and a smaller canal diameter, followed by the lumbar spine (20%), the cervical spine (9%), and the sacral spine (1%) [3,6]. In 70% of the cases, only one region of the spine is affected [11].

Any tumor can metastasize to the spine. However, the most common sites are the lung (24%), breast (24%), liver (12%), prostate (11%), and kidney (1%) [12]. In 10% of patients, spinal metastases are the first manifestation of an unknown primary tumor [6].

Spinal metastases are classified according to their location in relation to the dura mater [5]. Extradural metastases are much more common (>90%) due to their connection to the venous system. Intradural metastases account for about 5% and intramedullary metastases account for less than 1% [3,5,6,13].

Symptomatic presentations of spinal metastases can lead to a significant decrease in quality of life and functional status and an increase in mortality [3,8]. Chronic back pain is the most common initial symptom, requiring careful evaluation and screening for metastases in patients with a history of cancer [4,10]. Thoracic pain is particularly suspicious and should be investigated, as degenerative processes are more likely to occur in the cervical and lumbar regions [10]. Neurologic symptoms, such as weakness, sensory or gait disturbances, and bowel or bladder dysfunction, often develop weeks or months after the initial pain due to compression of the spinal cord or spinal nerves [12,14,15]. According to the literature, 10–20 % of patients with metastatic spinal cord compression will require treatment due to the extent of compression and the progression of neurological symptoms [15,16,17]. Additional potential manifestations include biomechanical instability, pathological vertebral fractures, and deformity [3,9,18].

The purpose of this study is to (1) assess symptom scores, (2) determine the diagnosis, (3) evaluate treatment modalities, (4) review prognostic scores, and (5) analyze management algorithms for spinal metastases to guide therapy selection.

## 2. Materials and Methods

### 2.1. PRISMA Statement

**Figure 1:**
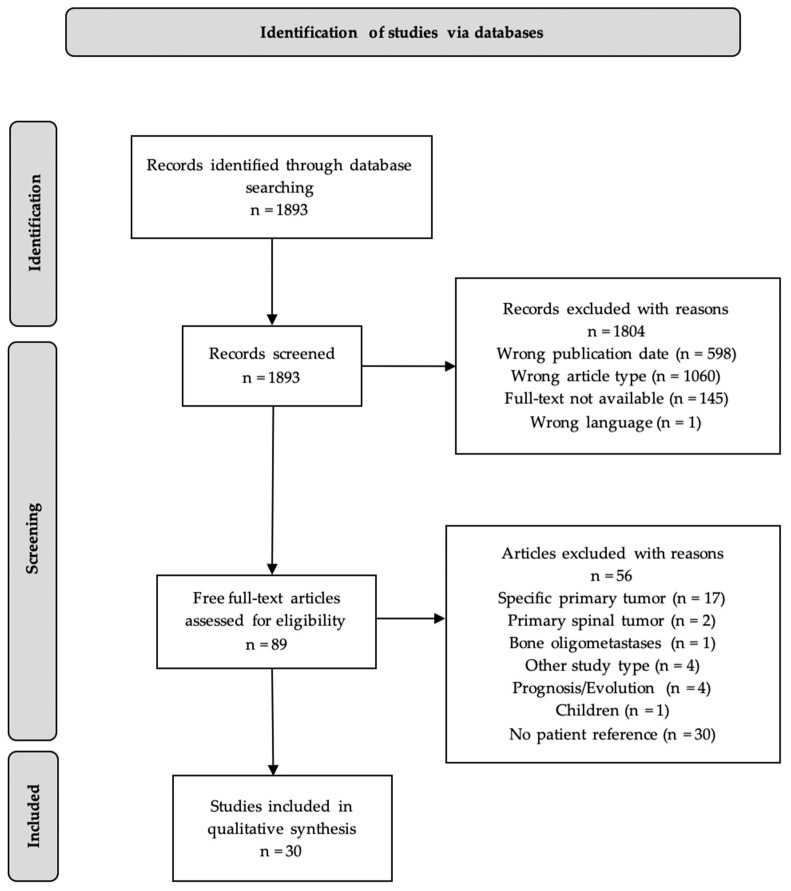
PRISMA flowchart of literature search and analysis [19].

The systematic review followed the recommendations of the Preferred Reporting Items for Systematic Reviews and Meta-Analyses (PRISMA), as shown in the flowchart in Figure 1 [19]. The protocol has not been registered.

### 2.2. Search Strategy

A thorough literature search was conducted in September 2022 using PubMed as the main database. To ensure a comprehensive review, a backward and forward cross-referencing process was also performed to identify potentially matching studies.

By applying PubMed filters for publication date from 1 January 2012 to 1 July 2022, English language, review article type, and free full-text availability, the number of relevant articles was reduced to 89 (Figure 1).

Titles and abstracts of the PubMed literature were initially searched using the following search string: spinal metastasis OR spinal metastases OR spine metastasis OR spine metastases AND treatment OR management, and a total of 1893 studies were identified.

### 2.3. Selection Process

These papers were then read and screened against predefined inclusion and exclusion criteria, as shown in Table 1, resulting in a final selection of 30 papers to be included in this review, whose characteristics are summarized in Table 2.

## 3. Results

### 3.1. Symptomatic Scores

#### 3.1.1. Pain

The most common initial symptom of spinal metastases is back pain, although the variety of possible causes, such as non-malignant back pain, may lead to delayed diagnosis or treatment [3,6,31,32]. It is important to rule out other potential differential diagnoses before initiating treatment [31,33].

Pain impairs quality of life; therefore, its management is of great importance and various conservative, pharmacologic, radiotherapeutic, and surgical options, as well as nerve blocks, are available [5,26,31]. Treatment efficacy should be assessed at three months post-treatment using the Brief Pain Inventory (BPI), with the Visual Analog Scale (VAS) and Numerical Rating Scale (NRS) remaining the most commonly used scoring systems [4,27].

#### 3.1.2. Neurological Deficits

Neurological status is graded on a five-point scale (a to e), as shown in Table 3 [23]. One cause of neurological deficits is epidural spinal cord compression (ESCC), which can be determined by MRI. The ESCC scale, also known eponymously as the Bilsky scale, classifies the severity of ESSC into low grade (grades from 0 to 1c) and high grade (grades 2 and 3) [6,14,15,27,32,33]. Patients with grade 1 ESSC and radiosensitive tumors, or grade 2–3 ESSC without neurological symptoms can be treated with radiotherapy alone [10,17,27,32]. In addition, the administration of radiotherapy in the treatment of low-grade ESCC is important to prevent the progression of the ESCC grade and the need for surgery [14].

#### 3.1.3. Spinal Stability

The Spinal Instability Neoplastic Score (SINS) is a recommended tool to assess spinal stability in the context of metastatic disease, to evaluate the risk of pathologic fracture, and to guide treatment decisions [6,9,11,14,34]. If the SINS score result is “stable”, radiotherapy alone may be sufficient [11,32]. In all other cases, surgical stabilization is indicated, either as a prophylactic measure before planned chemotherapy or radiation therapy or to prevent the progression of instability [5,6,11,17,32,34].

It is important to note that mechanical instability and neurological compression are independent indications for surgery [27,30].

### 3.2. Diagnosis

Early diagnosis of patients with neurological symptoms is crucial for optimal treatment choice, outcome of neurological symptoms, and improvement in patient life expectancy, as these symptoms are irreversible [11,15,16,26]. The average time from first symptoms to diagnosis of spinal metastases is four months and has a significant impact on quality of life [11,16].

Radiography is the most appropriate initial evaluation for back pain, but it is not useful for detecting bone metastases because more than 50% of the mineral density in the trabecular bone must be destroyed to be visible [3,5,26,35].

Computed tomography (CT) is superior to radiography in detecting spinal metastases because it provides a very good assessment of the trabecular and cortical bone, as well as the vertebral body, spinal canal and neurovascular structures [3,11,26]. However, CT cannot reliably exclude the presence of metastases with certainty as it has a sensitivity of 83% and a specificity of 85% [3,9].

Magnetic resonance imaging (MRI) is the gold standard for imaging and detecting spinal metastases with a sensitivity of 93–98.5% and a specificity of 97–100% [3,5,6,9,10,11,14,26]. It can determine the extent of ESCC and whether the cause of vertebral body collapse is benign or malignant [3,14].

National guidelines in the Netherlands outline specific diagnostic timelines for patients with cancer. For patients with localized back pain or unilateral radicular pain, MRI of the entire spine should be performed within two weeks and one week, respectively. For patients with bilateral radicular deficits or metastatic ESCC, MRI should be performed as soon as possible, ideally within 12 h. If a metastatic lesion is suspected, but the primary tumor remains unidentified, a thorax and abdomen positron emission tomography (PET-CT) scan and biopsy should be performed within one week of diagnosis [9].

Biopsy is the gold standard for definitive and accurate diagnosis and provides further information about the primary tumor if it is unknown [3,5,6,9,33]. It is also used for staging, treatment planning, and prognosis [3,26].

Other imaging modalities are gaining importance. Bone scintigraphy is pivotal for the assessment, staging, and management of bone metastases [3,10]. PET and single photon emission computed tomography (SPECT) provide critical insights into metabolic processes when MRI is inconclusive [35,36]. SPECT/CT has a sensitivity of 94% and a specificity of 71% for the detection of bone metastases [3].

### 3.3. Treatment Modalities

Treatment decisions are based on a comprehensive evaluation of risks, benefits, and expected survival [7,22,32]. The primary goals of treatment include pain control, spinal stabilization, improvement or prevention of neurologic symptoms, local control, and preservation of quality of life [6,8,14].

#### 3.3.1. Conservative Therapy

Patients with a life expectancy of less than three months are generally ineligible for surgery because the potential risks outweigh the benefits and should be treated with purely conservative therapy or a single fraction of conventional external beam radiation therapy (cEBRT) for symptom relief [5,7,10,12,33,37].

Conservative treatment includes exercise, behavioral therapy, physical therapy, and occupational therapy aimed at facilitating mobility, strengthening, and stretching muscles to improve daily function. However, a comprehensive rehabilitation program requires a risk-benefit assessment and regular clinical evaluation for mechanical axial loading and neurological deficits to prevent exacerbation of painful stimuli.

In addition, paracetamol and nonsteroidal anti-inflammatory drugs (NSAIDs) are the initial pharmacologic treatment for mild to moderate pain [31]. Opioids, including morphine, oxycodone, hydromorphone, and fentanyl, are the most effective medical treatment of last resort, often combined with other analgesics to minimize associated side effects [7,30,31,32]. Intrathecal or epidural opioid pumps may be considered when pain management is inadequate due to significant side effects with other routes [5,31,32].

Biologic pain is treated with dexamethasone, often in combination with radiotherapy [5,6,7,14]. Initiation of corticosteroid therapy within 12 h of the onset of compression symptoms has been shown to mitigate acute symptoms and to increase the number of patients with preserved ambulation one year after treatment [5,23,33]. Delay increases the risk of losing ambulation within one year by a factor of six [33].

The preferred choice of treatment for neuropathic pain is antiepileptic drugs, such as tricyclic antidepressants and gabapentin, although radiotherapy or surgical decompression is often required as the disease progresses [4,6,31,32].

#### 3.3.2. Palliative Therapy

Palliative care, including pain management by a pain specialist, is crucial for patients who are ineligible for radiation or surgery and have a short life expectancy [32]. It addresses the physical, psychological, social, and spiritual needs of the patient and family while respecting the patient’s wishes [9].

#### 3.3.3. Surgery

The indication for surgery depends on several factors, including life expectancy, surgery-related morbidity, overall health status, tumor histology, the balance of potential benefits and risks, and the probability of symptom improvement [5,7,18,33]. Surgical intervention following radiotherapy or chemotherapy is considered if neurological symptoms worsen, metastatic progression is observed, or if persistent high-grade ESCC shows no improvement [5].

Surgical procedures for spinal metastases have become increasingly established, with the study by Patchell et al. [38] significantly influencing surgical indications and goals [4,14,28]. This prospective, randomized, controlled trial of 101 patients, demonstrated superior outcomes for patients with radioresistant spinal metastases and spinal cord compression who underwent decompressive surgery followed by postoperative radiotherapy compared to those who received radiotherapy alone [38]. The primary endpoint of walking ability was significantly improved in the surgical group, with 84% of patients able to walk compared to only 57% in the radiotherapy group [38]. In addition, patients in the surgical group had significantly better recovery rates and reduced use of opioids and corticosteroids [38]. Both treatment groups received the same cEBRT radiation dose of 30 Gy in 10 fractions with a two-week postoperative interval [38]. The study recommended decompressive surgery followed by radiotherapy as the primary treatment option for patients with radioresistant metastatic ESCC, regardless of the presenting symptoms, given the significant risk of neurological complications or loss of ambulation associated with radiation therapy alone [38]. A further study showed that postoperative walking ability was significantly dependent on the preoperative lower limb muscle strength and the preoperative walking ability [18].

Studies have shown that postoperative survival is significantly influenced by preoperative neurological status, performance status, life expectancy as measured by the modified Tokuhashi score, and histologic response to postoperative radiotherapy [5,18]. Furthermore, surgery should only be considered if postoperative systemic therapy, possibly targeting genetic mutations, is available [5,18,33].

##### Decompression Surgery

Patients with significant spinal instability, acute symptomatic high-grade ESCC, and an increased risk of compression due to local progression or radioresistant tumors, regardless of the presence or severity of symptoms, are indicated for primary decompression surgery or separation radiosurgery to optimize postoperative stereotactic body radiation therapy (SBRT) [3,5,6,9,13,21,27,32]. Patients with acute neurologic deficits and sudden loss of ambulation due to acute ESCC, should also undergo immediate surgical decompression within 48 h of onset, in the absence of contraindications, which is the optimal treatment to improve the chances of recovery and preservation of neurologic function [11,16,18,23].

##### Minimal Invasive Surgery (MIS)

MIS is appropriate for patients with a life expectancy > 3 months, while open surgical techniques are reserved for patients with a life expectancy > 6 months, and aggressive surgical metastasectomy for local tumor control (Total En Bloc Spondylectomy—(TES)) is indicated only for patients with a projected survival > 2 years due to a significant postoperative mortality rate [8,15,33].

Percutaneous vertebral augmentation, such as vertebroplasty or kyphoplasty, possibly in combination with fenestrated (cemented) and pedicle screws, is considered the preferred surgical approach for spinal stabilization and when management with medication has failed to provide adequate pain relief [3,10,17,29,32]. Prophylactic vertebral augmentation is recommended when the only characteristics present are a “lytic lesion” and “vertebral body collapse of approximately 50%”, as this already indicates an increased risk of fracture [17].

#### 3.3.4. Radiation Therapy

Primary radiotherapy is recommended for patients with a radiosensitive tumor without spinal cord compression or instability to achieve local tumor control [5,6,10,15,32]. Additional indications include tumor expansion-related bone pain, isolated ESCC without bone involvement, symptom relief in ineligible surgical candidates with a life expectancy < 3 months or severe neurological deficits lasting more than 48 h [3,30,32]. This suggests that timely initiation of radiotherapy may avoid surgical intervention [5].

Given the technological advances, minimal side effects, and effective symptom relief, radiotherapy plays a pivotal role in the treatment of spinal metastases [4].

##### Stereotactic Body Radiation Therapy (SBRT)

Table 4 outlines the inclusion and exclusion criteria for patients undergoing SBRT [20,25].

Improved overall survival after radiotherapy has been observed, particularly in women, in patients with good performance status, in those undergoing postoperative radiotherapy, and in the presence of a solitary metastasis [25].

SBRT should not be considered as a substitute for surgery, as surgery may result in more rapid neurological improvement. In addition, SBRT planning typically takes one to two weeks, and treatment should be initiated within 48 h of the onset of neurological deficits [20].

It is important to consider the tumor histology when planning treatment, as radiosensitive tumors should be treated with radiotherapy first and should be evaluated for existing symptoms [18].

##### Radiofrequency Ablation (RFA)

Percutaneous thermal ablation, such as RFA, represents a viable alternative for relieving compression-induced symptoms in patients ineligible for surgery [30]. Ablation is often combined with radiation therapy and vertebral augmentation, a combination recommended for the treatment of asymptomatic or painful pathologic vertebral fractures [7,29,30,32,33]. Stand-alone ablation is recommended for patients with uncomplicated, asymptomatic, or painful spinal metastases who have a life expectancy of more than six months, few visceral metastases, and a favorable performance status [30].

### 3.4. Prognostic Factors

The prognosis of patients with spinal metastases is primarily determined by three key factors: quality of life, performance status, and life expectancy [6,15,37,39]. Treatment selection and efficacy are significantly influenced by a patient’s life expectancy [11,14,15,16].

#### 3.4.1. Life Expectancy

The modified Tokuhashi score, or the Tomita score, used to estimate life expectancy, influences the treatment decision [5,10,11].

Depending on the primary tumor and radiosensitivity, radiotherapy, particularly single-fraction cEBRT or SBRT, has been shown to be an effective treatment for pain in patients with spinal metastases without spinal cord compression and a life expectancy of < 3 months; however, a higher radiation dose or a multi-fraction scheme did not demonstrate superior efficacy for pain relief [3,4,33]. If pain persists after initial radiotherapy, a second course of radiotherapy can be given after four weeks [4,27].

A limitation of life expectancy models is their tendency to identify palliative care candidates rather than surgical candidates [6,22]. Therefore, a comprehensive assessment of the patient’s overall clinical condition is crucial to determine the optimal treatment approach, as surgery may be recommended if a good prognosis is given [33,37].

For patients with longer life expectancy, a combined approach of surgery and radiation therapy is recommended, prioritizing improvement of quality of life, mechanical stability, and pain relief rather than tumor removal [7,8,9,37].

#### 3.4.2. Quality of Life

The Spine Oncology Study Group Outcome Questionnaire (SOSG-OQ) and the Patient Reported Outcomes Measurement Information System (PROMIS) were designed to assess quality of life. The SOSG-OQ was developed specifically for patients with spinal metastases and is superior to PROMIS [28].

#### 3.4.3. Performance Status

**Figure 2:**
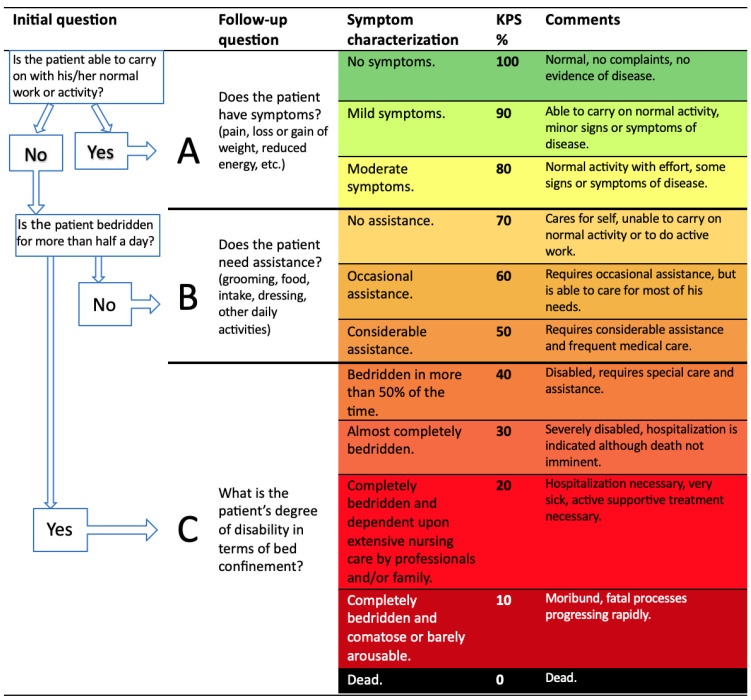
Proposed algorithm system for the evaluation of the KPS. (In this traffic light model, green indicates no symptoms. As the patient's condition worsens, the color shifts through a spectrum until it reaches black, which indicates death) [39].

The Karnofsky Performance Status (KPS) and the Eastern Cooperative Oncology Group Performance Scale (ECOG-PS) are used to assess patient performance status, as they allow the assessment of an individual’s ability to cope with daily activities through a series of questions, with the KPS being more accurate. In Figure 2, Péus et al. propose an algorithmic system for the evaluation of the KPS. The initial assessment includes yes/no answers to two to three questions to determine the functional status of the patient: A, B and C. Further questions refine the classification into 11 derivative KPS values from 100% (indicating no symptoms or signs of illness) to 0% (indicating death) with further suggestive clarification provided in follow-up questions (in round brackets). Symptom characterization in this framework is based on the work of Kasnofsky and Burchenal [28,39].

### 3.5. Management Algorithms for Spinal Metastases

#### 3.5.1. NOMS

The acronym NOMS, which includes four pillars including neurology, oncology, mechanical stability, and systemic condition, was proposed in 2013 as a management framework for spinal metastases [5,13,14,21,22,23,32,33]. The decision-making algorithm, frequently cited in several included studies, aims to optimize treatment selection among surgical stabilization or separation, cEBRT, SBRT, and pharmacologic modalities for each patient [6,13,21,24].

Neurological evaluation is based on clinical neurological status and radiologic assessment of ESCC severity using the Bilsky score [14,32,33].

Oncologic considerations include tumor histology and the response to radiation, especially to cEBRT [14,16,23,32].

Mechanical stability is assessed according to the SINS criteria, with instability alone already being an indication for surgery, regardless of ESCC severity [16,21,32,33]. It is important to identify instability, because radiotherapy alone may exacerbate further progression; therefore, cement augmentation with or without pedicle screws is recommended [6,23].

A thorough assessment of the patient’s systemic condition, including life expectancy, comorbidities (particularly pulmonary and cardiac disease), the extent of systemic disease, performance status, and the potential risks or benefits of the treatment, is essential to determine the patient’s ability to tolerate a surgical or more aggressive treatment strategy [14,32,33,40]. Because the spinal metastases’ treatment is still a palliative approach, it is essential to assess whether the patient’s ability to withstand anesthesia and extubation, as well as large amounts of fluids or blood transfusions [10,14].

**Figure 3:**
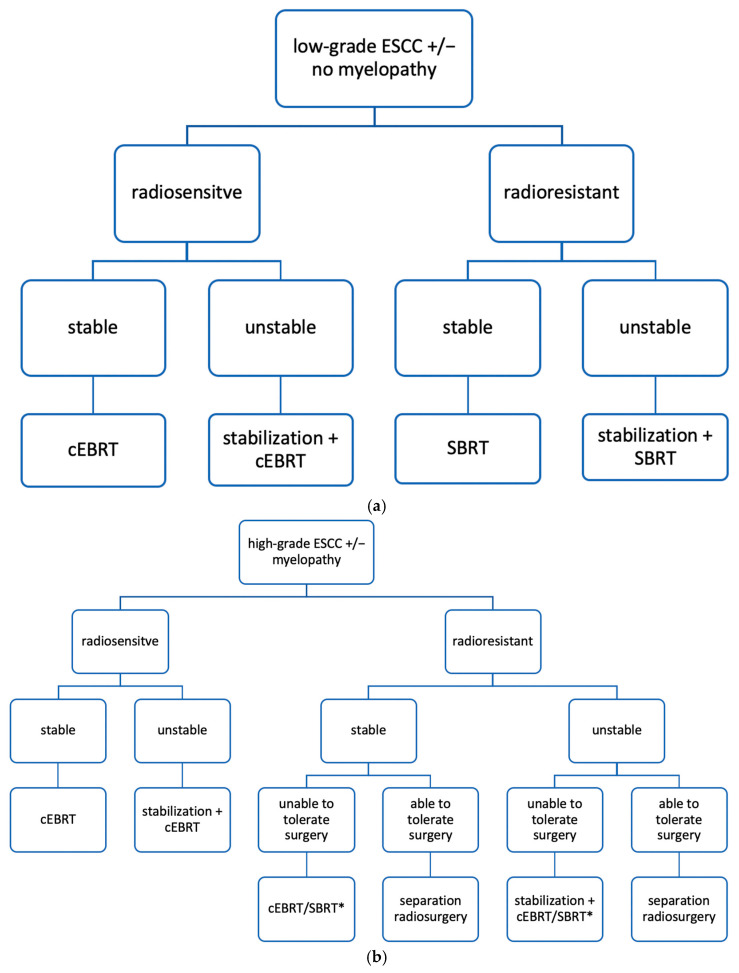
NOMS decision framework divided into: (**a**) low-grade ESCC; (**b**) high-grade ESCC [14,16,21,32]. * Given that surgical separation is not a viable option and the high complication rate associated with SBRT alone in high-grade ESCC, cEBRT is recommended as the initial treatment, with SBRT reserved for cases without improvement [21,32].

Figure 3 shows the NOMS algorithm, divided into low-grade and high-grade ESCC.

#### 3.5.2. LMNOP

The LMNOP algorithm considers localization (L), mechanical instability (M), neurology (N), oncology (O), performance status, prognosis, patient wishes, and previous therapy response (P) [9,22].

Localization is crucial for selecting the appropriate surgical approach for decompression and stabilization. Not seldomly, multiple levels of the spine may be affected, necessitating identification and treatment of the symptomatic vertebra [22].

Mechanical instability is identified by using SINS, with vertebral augmentation recommended for patients with “indeterminate instability” and additional stabilization with pedicle screws for patients with “instability” [22].

Neurology refers to the study by Patchell et al. [38], which demonstrated the efficacy of surgical decompression combined with postoperative radiotherapy to improve outcomes in patients with neurologic deficits [22].

Oncology, including histology of the primary tumor, determines radiation modality and life expectancy [22].

Compared to NOMS, LMNOP incorporates the number and location of metastases, and the response to prior therapy [22].

#### 3.5.3. Other Algorithms

The Harrington Classification is a simple decision-making algorithm that divides patients into five categories based on stability and neurological symptoms to guide further therapeutic interventions. Categories 1 and 2, characterized by the absence of neurological symptoms with or without instability, should be treated with systemic or radiation therapy. Conversely, patients in categories 4 or 5, who have instability with pain and/or neurological symptoms, should undergo surgical intervention. Category 3 includes patients with neurological symptoms but no evidence of instability, in whom several therapeutic approaches may be beneficial. For patients with loss of ambulation, urgent surgical decompression within 48 h is indicated [32].

Of the thirty studies reviewed, four have proposed treatment algorithms, but none have been independently validated [5,15,16,33]. The algorithm proposed by Conti et al. is based on the performance status and suggests palliative care for patients with a KPS below 40%, as shown in Figure 2. In the absence of contraindications, treatment recommendations depend on the presenting symptoms and tumor histology, considering options such as radiotherapy, surgical stabilization, decompression, or separation radiosurgery [15].

A study by Kurisunkal et al. differentiates between the presence or absence of ESCC after the diagnosis of metastatic spinal lesions. For patients without ESCC with persistent bone pain, medical therapy followed by radiotherapy is recommended. In contrast, in the absence of pain, a watch-and-wait approach is indicated. In the presence of ESCC, stability and histology determine further management. Accordingly, radiation therapy alone, surgical stabilization, or decompression followed by radiotherapy are recommended [5].

The treatment algorithm by Jaipanya et al. begins with MRI-based diagnosis of symptomatic spinal metastases, with PET/CT and biopsy being indicated for unknown primary tumors [9,33]. The first step is to determine the immediate need for corticosteroids and the optimal timing of surgery based on symptom severity. Further surgical management depends on life expectancy, with preoperative embolization considered for hypervascular tumors. MIS is recommended for patients with a life expectancy of more than three months, open surgery for patients with a life expectancy of more than six months, and TES for patients with a life expectancy of more than two years. Postoperative radiotherapy is recommended after any surgical procedure. For patients who are ineligible for surgery, palliative radiotherapy is recommended. Radiation therapy with cEBRT is recommended for radiosensitive tumors, while separation radiosurgery with SBRT is used for radioresistant tumors [12].

## 4. Discussion

Current concepts in the management and treatment of spinal metastases are primarily symptom driven. Therefore, a pain diary should be maintained until adequate pain control is achieved [41].

While the NRS and VAS are the most commonly used pain measurement scales, the BPI is internationally recommended for the assessment of chronic pain [41,42,43].

The SINS score requires CT data to assess stability. In contrast, MRI is the gold standard for diagnosis of suspected spinal metastases and assessment of ESCC.

Neurological deficits and spinal cord compression are identified by a comprehensive clinical examination in conjunction with imaging. The Bilsky score categorizes spinal cord compression in cases of spinal metastases. Neurological deficits are documented separately according to the neurological grades shown in Table 3 [23,32].

A multidisciplinary team, including oncologists, radiation oncologists, radiologists, orthopedic spine surgeons or neurosurgeons, anesthesiologists, pain specialists, primary care physicians, rehabilitation team, nurses, physiotherapists, and others, ensures optimal diagnosis and an individualized treatment plan for patients with spinal metastases, taking advantage of the latest advances in each medical discipline [5,6,9,11,14,30,32,33]. In most cases, a palliative approach is followed with the goal of relieving pain and maintaining stability or neurological function through local control [5,7,10,21]. Subsequent treatment is determined by evaluation of stability, neurologic deficits, pain status, life expectancy, performance status, and primary tumor histology [15,30].

The decision regarding further treatment depends largely on histologic clarification by targeted biopsy, determination of prognostic factors, and evaluation of the patient’s wishes. Quality of life, performance status, and life expectancy are important prognostic factors, although their determination is subject to certain research limitations and therefore can only be an estimate, as it is not possible to account for unexpected events.

Several therapeutic options are available, including conservative, systemic, surgical, and radiation therapy [10,21]. Advances in targeted therapies, radiotherapy, MIS, and combined treatment have significantly improved the management of spinal metastases. Recently, separation radiosurgery, which integrates surgical decompression with SBRT, has emerged as a promising treatment option.

The SORG nomogram is used to estimate the probability of survival for patients with spinal metastases at 3 and 12 months, regardless of any pending therapy. In contrast, the modified Tokuhashi score and the Tomita score are used to calculate life expectancy after surgery to facilitate treatment decisions [5,6,7,14,22,32,33,37]. While both scores are effective in identifying patients with poor prognosis, the modified Tokuhashi score provides more accurate predictions for moderate and good prognosis [37]. It should be noted that these prognostic tools consider the full range of potential treatment options and are primarily used for patients who require palliative care rather than those who may benefit from surgery. Before any intervention, it is critical to weigh the potential benefits and risks of surgical and other treatment options.

Current management algorithms for spinal metastases include the NOMS decision framework and the LMNOP. Four of the included papers [5,15,16,33] developed a treatment decision algorithm, with Jaipanya et al. [33] presenting the most recent algorithm.

### Review Limitarions

A large number of studies were excluded from the systematic review, due to incorrect publication dates, unsuitable article types or the lack of availability of full-text articles.

The heterogeneity of the reviewed studies in terms of study design, populations, available treatments and their protocols and sample sizes represents a limitation of this study.

## 5. Conclusions

Spinal metastases are a clinical challenge and have a significant impact on patients’ quality of life and life expectancy. Early diagnosis through a comprehensive clinical examination that identifies symptoms, neurological deficits, and spinal cord compression is crucial. MRI is the gold standard for suspected spinal metastases. A multidisciplinary team approach is essential to ensure optimal management. Treatment decisions should be individualized, could be guided by frameworks such as NOMS and LMNOP, and consider factors such as neurological status (as assessed by the Bilsky score), mechanical stability (as assessed by the SINS score), tumor histology, life expectancy (as assessed by scores such as Tomita and Tokuhashi), and patient wishes. Treatment options include conservative therapy, surgery (including MIS), and radiotherapy (including SBRT) with the primary goals of pain relief and the maintenance of stability and neurological function through local control. Further research should focus on evaluating different treatment options in combination with a symptomatic or prognostic scoring system to optimize treatment strategies. The use of apps for data collection and analysis will be instrumental in advancing research and providing a more detailed understanding of the implications associated with score results as well as standardized protocols to improve patient outcomes and quality of life.

## Figures and Tables

**Table 1:** Inclusion and exclusion criteria.

Inclusion Criteria
Identification of eligible patients
Treatment algorithm/selection/comparison for spinal metastases
Management and treatment of stability
Management and treatment of neurological symptoms
Management and treatment of pain
**Exclusion Criteria**
Primary tumor of the spinal metastasis as the main component
Primary brain/spine tumor
Bone metastases in general as the main component
Prognosis, evolution, future outlook
Children
No patient reference, mainly technical data on treatment options
Other reasons

**Table 2:** Study characteristics of the included publications.

Study	Year	Region	Country	Journal	Study Type	LoE *
Laufer et al. [10]	2012	North America	USA	Cancer Control	Review	5
Husain et al. [20]	2013	North America	USA	CNS Oncol	Review	5
Anwar et al. [17]	2013	North America	USA	CNS Oncol	Review	5
Moussazadeh et al. [21]	2014	North America	USA	Cancer Control	Review	5
Ivanishvili et al. [22]	2014	North America	Canada	Global Spine J	Review	5
Ryu et al. [23]	2015	North America	USA	Radiat Oncol J	Review	5
Barragan-Campos et al. [3]	2015	North America	Mexico	Rev Invest Clin	Review	5
Joaquim et al. [6]	2015	South America	Brazil	Arq Neuropsiquiatr	Review	5
De Moraes et al. [13]	2016	South America	Brazil	Clinics (Sao Paolo)	Review	5
Zuckerman et al. [24]	2016	North America	USA	Spine (Phila Pa 1976)	Review	5
Huo et al. [25]	2017	Australia	Australia	Surg Neurol Int	Review	5
Curtin et al. [26]	2017	Europe	Ireland	Orthop Surg	Review	5
Szendroi et al. [16]	2017	Europe	Hungary	EFORT Open Rev	Review	5
Pennington et al. [12]	2018	North America	USA	Ann Transl Med	Review	5
Groenen et al. [9]	2018	Europe	The Netherlands	Cancer Treat Rev	Review	5
Le et al. [4]	2018	North America	USA	Cureus	Review	5
Zeng et al. [27]	2019	North America	Canada	Front Oncol	Review	5
Yahanda et al. [28]	2019	North America	USA	Ann Transl Med	Review	5
Conti et al. [15]	2019	Europe	Germany	Front Oncol	Review	5
Barzilai et al. [29]	2020	North America	USA	Neurosurg Clin N Am	Review	5
Kurisunkal et al. [5]	2020	Asia	India	Indian J Orthop	Review	5
Tomasian et al. [30]	2020	North America	USA	Semin Intervent Radiol	Review	5
Newman et al. [14]	2020	North America	USA	Neurooncol Pract	Review	5
Sarma et al. [31]	2021	North America	USA	Front Pain Res (Lausanne)	Review	5
Zaveri et al. [32]	2021	Asia	India	Indian J Orthop	Review	5
Kato et al. [8]	2021	Asia	Japan	Oncologist	Review	5
Dhamija et al. [18]	2021	Europe	UK	J Clin Orthop Trauma	Review	5
Serratrice et al. [11]	2022	Europe	France	Front Oncol	Review	5
Jaipanya et al. [33]	2022	Asia	Thailand	J Int Med Res	Review	5
Giammalva et al. [7]	2022	Europe	Italy	Life (Basel)	Review	5

* LoE: Level of evidence.

**Table 3:** Neurological grade [23].

a	No symptoms
b	A focal minor symptom
c	Functional paresis with muscle strength of 4 or 5 out of 5, due to either nerve root or spinal cord compression
d	Non-function paresis with muscle strength of 3 or less out of 5, due to either nerve root or spinal cord compression
e	Complete paresis or urinary and rectal incontinence

**Table 4:** Inclusion and exclusion criteria for SBRT [20,25].

Inclusion Criteria
A maximum of three contiguous or non-contiguous spinal segments
No or minimal spinal instability (SINS 0 to 6)
No or minimal epidural disease (Bilsky 0 to 1)
Radioresistant histology
Age > 18 years
Karnofsky Performance Scale (KPS) minimum of 40
Life expectancy greater than three months
Previous cEBRT
Postoperative setting
**Exclusion Criteria**
ESCC or cauda equina compression requiring decompression (Bilsky 2–3)
Instability requiring stabilization (SINS 7–18)
Inability to remain still and tolerate treatment
Contraindication to undergo a full spinal MRI and/or CT myelogram
Systemic radionuclide delivery within 30 days prior to SBRT
cEBRT within 90 days prior to SBRT

## Data Availability

No new data were created or analyzed in this study.
